# Interaction Between Peroxisome Proliferator-Activated Receptors and Cannabidiol in the Gut of Chickens Applied to Different Challenge Conditions

**DOI:** 10.3390/ijms252111398

**Published:** 2024-10-23

**Authors:** Dominika Szkopek, Marta Mendel, Misza Kinsner, Bartosz Fotschki, Jerzy Juśkiewicz, Krzysztof Kozłowski, Paulius Matusevičius, Paweł Konieczka

**Affiliations:** 1 Department of Animal Nutrition, The Kielanowski Institute of Animal Physiology and Nutrition, Polish Academy of Sciences, Instytucka 3, 05-110 Jabłonna, Poland; m.kinsner@ifzz.pl; 2 Division of Pharmacology and Toxicology, Institute of Veterinary Medicine, Warsaw University of Life Sciences, Ciszewskiego 8, 02-786 Warsaw, Poland; marta_mendel@sggw.edu.pl; 3 Institute of Animal Reproduction and Food Research, Polish Academy of Sciences, Tuwima 10, 10-748 Olsztyn, Poland; b.fotschki@pan.olsztyn.pl (B.F.); j.juskiewicz@pan.olsztyn.pl (J.J.); 4 Department of Poultry Science and Apiculture, University of Warmia and Mazury in Olsztyn, Oczapowskiego 5, 10-719 Olsztyn, Poland; kristof@uwm.edu.pl; 5 Department of Animal Nutrition, Lithuanian University of Health Sciences, Tilzes 18, LT-47181 Kaunas, Lithuania; paulius.matusevicius@lsmu.lt

**Keywords:** cannabidiol, proliferator-activated receptors, *Clostridium perfringens*, necrotic enteritis, colibacteriosis, *E. coli* LPS, gastrointestinal tract, broiler chickens

## Abstract

Peroxisome proliferator-activated receptors (PPARs) are important targets for cannabidiol (CBD), which mediate many of its biological actions. The hypothesis of the present research assumed that PPARs affect the gut response to different challenge factors in chickens (*C. perfringens* vs. lipopolysaccharides (LPS) from *E. coli*), and that CBD can mediate the pathways of this response. The study proved that CBD and the challenge factors significantly affect the expression level of PPARα (*p* = 0.001) and selected genes determining gut barrier function. A positive correlation was demonstrated between PPARs and genes involved in the formation of tight junctions, immune, and oxidative stress responses in chickens. Dietary supplementation with CBD actively mediated the expression rate of PPARs, but the mechanism of interaction between CBD and PPARs was different depending on the stress factor used. The addition of CBD to the birds’ diets did not contribute to reducing intestinal permeability under induced stress conditions nor cause stress, as indicated by the absence of elevated blood cortisol and endotoxin levels. CBD also supported the mechanisms of protecting intestinal cells from the cytotoxic effects in a *C. perfringens* challenge through the levels of genes involved in oxidative stress. This study indicates the importance of research toward understanding the mechanisms of PPARs as a target for enhancing intestinal barrier function, provides new results on the biological action of CBD in chickens, and shows a constant PPAR association with the jejunum mucosa of birds.

## 1. Introduction

The poultry industry, mainly the production of broiler chickens, is one of the fastest-growing sectors of livestock production worldwide. Broiler chickens are undergoing intensive selection, mainly due to their fast growth rate and good feed utilization. Consequently, modern lines of broiler chickens will reach slaughter weight at 35 days of age. The negative consequences of such a rapid growth rate include a deterioration in overall welfare, reduced resistance to stress factors, and increased susceptibility to infection. The natural result of such a situation is the increasing use of veterinary drugs in broiler housing. The European Medicines Agency (EMA) has noted an increasing trend in the use of antibiotics (per kilogram of body weight) to treat birds, not only due to the increasing pace of production, but also to the growing health problems of commercially kept birds [1,2]. The use of bioactive compounds, such as natural additives, in poultry nutrition is one potential alternative to the antibiotic-based approach.

The hypothesis of the present research assumed that peroxisome proliferator-activated receptors affect the gut response to different challenge factors in chickens, and cannabidiol can mediate the pathways of this response. Cannabidiol is one of the main pharmacologically active phytocannabinoids of the *Cannabis sativa* L. plant. Although cannabidiol has been used in several animal studies, the lack of research on CBD use in poultry is notable [3]. CBD has no psychoactive effects but has many demonstrated beneficial properties including anti-inflammatory, antimicrobial, and antioxidant properties [4]. The main targets of CBD activity are nuclear receptors such as the peroxisome proliferator-activated receptors [5]. *PPAR*s are a family of nuclear receptors with three subtypes: *PPARγ*, *PPARα*, and *PPARβ/δ*. All three *PPAR* isotypes have a strong impact on various aspects of the physiology of the immune system and exhibit potent anti-inflammatory properties that have been detected in several cell types including immune cells, muscles, adipose tissues, and the brain. Thus, CBD, as a *PPAR* agonist, promotes significant pharmacological effects [5,6,7]. Cannabidiol not only activates *PPAR*s, but also affects their expression in the body [8]. Peroxisome proliferator-activated receptors stimulate the expression of many genes and mediate glucose homeostasis, lipid metabolism, cell fate, inflammation, and immune responses [9,10]. Growing evidence suggests that *PPAR*s and their natural agonists control the inflammatory response and barrier function of endothelial and epithelial cells [11,12,13,14]. The activation of *PPAR*s enhances barrier function and upregulates tight junction (TJ) protein expression in intestinal, brain, pulmonary, and urothelial epithelial cells (ECs) [11,12,15,16]. Cannabinoids also exert their pharmacological effects by acting on the cannabinoid (CB) receptors of the endocannabinoid system. The endocannabinoid system, consisting of endogenous ligands and receptors (CB1, CB2), plays a key role in controlling many physiological processes including gastrointestinal motility, food intake, intestinal inflammation, and cell proliferation in the gut, making it a potential therapeutic target for pathophysiological processes including pain and inflammation [17,18,19]. In the endocannabinoid system, the role of intercellular mediators is regulated by fatty acids or their metabolically active derivatives. Interestingly, *PPAR*s have large ligand binding domains and can be activated by several ligands of different chemical structures such as the aforementioned fatty acids, and they oxidize metabolites of linoleic acid such as eicosanoids or polyunsaturated fatty acids (e.g., arachidonic acid, which is a precursor to, for example, prostaglandins) and many plant extracts [20,21,22,23]. Studies suggest that cannabinoids inhibit the biosynthesis of pro-inflammatory prostaglandins (PGEs) through the acetylation of cyclooxygenase-2, and due to the similarity of PGEs to endocannabinoid structures, there is an interaction between metabolic pathways [24,25]. Due to the direct link between the initial inflammatory pathway and the loss of integrity of intracellular junctions, CBD, and its effects on *PPAR*s, may exert a modulating effect on the intestinal barrier and can be an effective intervention for treating inflammation [11,12,15,16,26,27,28].

The importance of bioactive nutrients in promoting and modulating gastrointestinal tract (GIT) functions indicates the potential for the development of new and effective applications in disease prevention in birds [29]. Klasing [30] reported that the effective nutritional modulation of resistance in birds is possible due to the substrate functions of nutrients throughout several mechanisms, for example: (i) direct regulation and influence on the development of the immune system, (ii) supply of substrates to the immune system and nutritional immunity, (iii) changes to the hormonal milieu, and (iv) physical and chemical actions on the intestines and reduction in pathology. These findings support the concept that the nutritional modulation of GIT functions may occur due to the regulatory action of bioactive nutrients. The gastrointestinal tract, specifically the gut barrier, is the first line of defense against antigens, toxins, and pathogens and largely determines the immune response. The intestinal epithelium is a selective barrier that allows the transport of various ions and nutrients. The intestine defense mechanisms including physical, chemical and immunological barriers are essential for maintaining body homeostasis [31]. Although the function of the intestinal barrier varies depending on the intestinal segment, the barrier is an essential component of the innate immune response [32]. The integrity of the intestinal barrier is maintained through transmembrane tight junction proteins. TJ proteins are crucial for the paracellular permeability of the epithelium, thus preventing harmful substances from entering the bloodstream [33].

One of the most common problems in chickens is necrotic enteritis (NE), caused by *Clostridium perfringens* (*C. perfringens*), which is an anerobic bacterium, and colibacteriosis, caused by *Escherichia coli* (*E. coli*). NE and colibacteriosis are estimated to be responsible for high economic losses in the poultry industry due to increased mortality and morbidity as well as impaired digestion and the malabsorption of nutrients, resulting in reduced growth rates and a worsened feed conversion ratio [34,35,36,37,38]. Due to the prohibition on the preventive use of antibiotic growth promoters and antibiotics in the European Union in 2006 (Regulation No. 1831/2003), there has been a rise in the incidence of *C. perfringens* and *E. coli* infections in broiler flocks. The acute form of NE leads to increased mortality. Birds with the subclinical form of NE do not show any clinical signs, but there is ongoing damage to the intestinal mucosa. Although many *E*. *coli* strains are harmless commensals, a subset has acquired the ability to cause extraintestinal or intestinal diseases. Enterotoxic strains of *E. coli* lead to acute enteritis of the small intestinal mucosa, which manifests as diarrhea. Moreover, broilers with mild NE and those infected with enterotoxigenic strains of *E. coli* pose a potential threat as a vector of bacteria and/or the toxins they produce into the food chain [35,39,40,41]. Therefore, the broiler industry will continue to pose a serious threat to human health due to its role as a vector of zoonotic diseases and its contribution to the rise in antibiotic resistance due to inappropriate drug use. For these reasons, there is an urgent need for natural nutritional supplements that will also not contribute to the problem of antibiotic resistance. Effective infection control strategies can be achieved by identifying the interactions occurring in the avian gastrointestinal tract and understanding the interaction between the functional state of the gastrointestinal tract and bioactive nutrients such as cannabidiol.

## 2. Results

### 2.1. CBD-, LPS-, and C. perfringens-Mediated Changes in the Transcript Levels of Selected Genes

#### 2.1.1. The Relative mRNA Expression Levels of *PPAR*s in the Jejunum of Chickens

**Figure 1:**
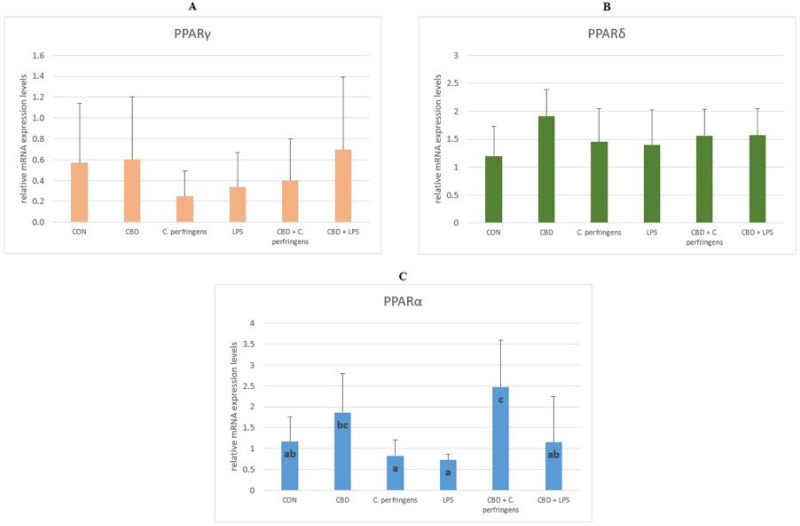
The relative mRNA expression levels (normalized to the glyceraldehyde-3-phosphate dehydrogenase and β-actin expression levels as the most accurate endogenous control gene) of (**A**) peroxisome proliferator-activated receptor delta (*PPARδ*), (**B**) peroxisome proliferator-activated receptor gamma (*PPARγ*), and (**C**) peroxisome proliferator-activated receptor alpha (*PPARα*) in the jejunum tissue of chickens. The chickens were fed a control diet (CON), CON supplemented with 30 g of hemp extract/kg diet (CBD), CON diet and subjected to *E. coli* LPS and *C. perfringens* challenge (LPS and *C. perfringens*), and CON diet supplemented with 30 g of hemp extract/kg diet and subjected to *E. coli* LPS and *C. perfringens* (CBD + LPS and CBD + *C. perfringens*). Significant differences are indicated by different letters (*p* < 0.05). The error bars indicate the pooled standard deviations in each dietary treatment group for the eight chickens.

The *PPARδ* and *PPARγ* mRNA expression levels were not significantly different between the study groups (*p* > 0.05). However, compared to the CBD group, the expression of PPARα was significantly lowered in both challenged groups (with *C. perfringens* or *E. coli* LPS) (*p* = 0.001). It should be noted that dietary supplementation with CBD in the challenged groups significantly increased the *PPARα* expression rate (*p* = 0.001). In all groups, the expression rate of this gene was at the same level or higher compared to that of the control (Figure 1).

#### 2.1.2. The Relative mRNA Expression Levels of Genes Involved in the Formation of Tight Junctions in the Gut Tissue of Chickens

**Figure 2:**
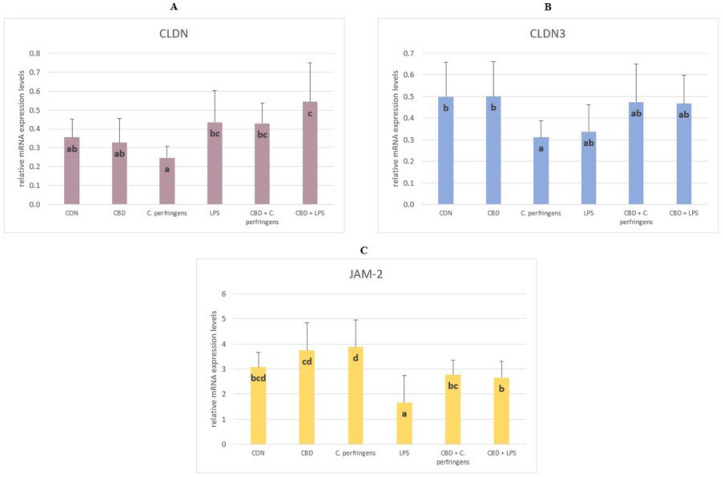
The relative mRNA expression levels (normalized to the glyceraldehyde-3-phosphate dehydrogenase expression and β-actin levels as the most accurate endogenous control gene) of (**A**) claudin (*CLDN*), (**B**) claudin 3 (*CLDN-3*), and (**C**) junctional adhesion molecule 2 (*JAM-2*) in the jejunum tissue of chickens. The chickens were fed a control diet (CON), CON supplemented with 30 g of hemp extract/kg diet (CBD), CON diet and subjected to *E. coli* LPS and *C. perfringens* challenge (LPS and *C. perfringens*), and CON diet supplemented with 30 g of hemp extract/kg diet and subjected to *E. coli* LPS and *C. perfringens* (CBD + LPS and CBD + *C. perfringens*). Significant differences are indicated by different letters (*p* < 0.05). The error bars indicate the pooled standard deviations in each dietary treatment group for the eight chickens.

The mRNA expression level of *CLDN* was significantly higher in the CBD + LPS and CBD + *C. perfringens* groups compared to the infected group without CBD addition (*p* = 0.003). The *CLDN-3* levels were significantly decreased in the *C. perfringens*-infected group compared to the CBD and control group (*p* = 0.048). The infection did not negatively affect the *CLDN-3* levels in the infected groups with CBD addition. The level of *JAM-2* was significantly lower in the LPS group compared to the control groups with and without CBD supplementation and the infected group with CBD supplementation, and was significantly increased in the *C. perfringens* group compared to the CBD + *C. perfringens* group (*p* = 0.001) (Figure 2).

#### 2.1.3. The Relative mRNA Expression Levels of Genes Involved in Gut Mucosal and Gastrointestinal Tract Defense in Inflammation

**Figure 3:**
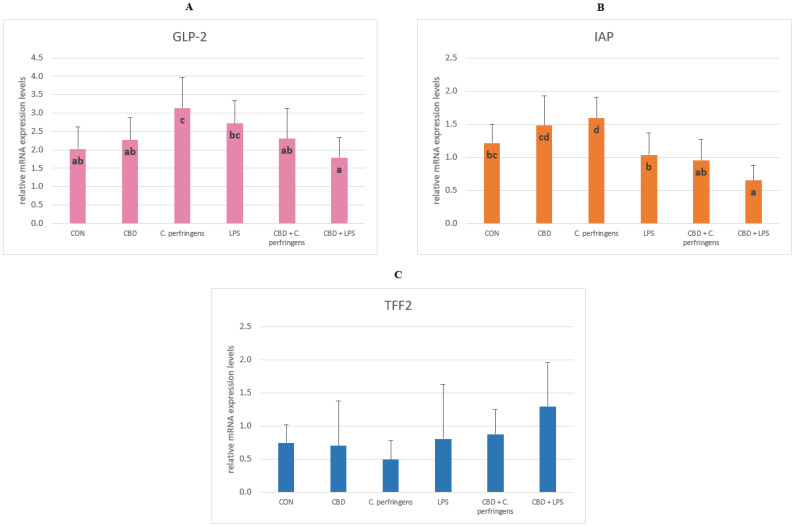
The relative mRNA expression levels (normalized to the glyceraldehyde-3-phosphate dehydrogenase and β-actin expression levels as the most accurate endogenous control gene) of (**A**) glucagon like peptide 2 (*GLP-2*), (**B**) intestinal alkaline phosphatase (*IAP*), and (**C**) trefoil factor 2 (*TFF2*) in the jejunum tissue of chickens. The chickens were fed a control diet (CON), CON supplemented with 30 g of hemp extract/kg diet (CBD), CON diet and subjected to *E. coli* LPS and *C. perfringens* challenge (LPS and *C. perfringens*), and CON diet supplemented with 30 g of hemp extract/kg diet and subjected to *E. coli* LPS and *C. perfringens* (CBD + LPS and CBD + *C. perfringens*). Significant differences are indicated by different letters (*p* < 0.05). The error bars indicate the pooled standard deviations in each dietary treatment group for the eight chickens.

The expression level of *GLP-2* was significantly lower in the CBD-infected groups compared to the control-infected groups (*p* = 0.005). *GLP-2* levels between the CON and CBD groups were not significantly different. The level of *IAP* was significantly higher in the infected groups without the additive compared to the infected groups with CBD, and its level was significantly lower in the CBD + LPS group compared to all tested groups (*p* = 0.001). The expression level of the *TFF2* gene did not differ between the dietary treatment groups (Figure 3).

#### 2.1.4. The Relative mRNA Expression Levels of Genes Involved in the Oxidative Stress

**Figure 4:**
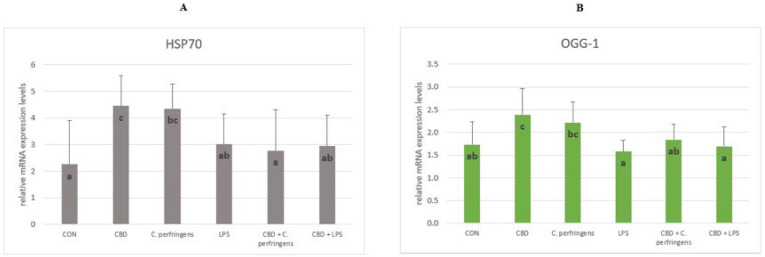
The relative mRNA expression levels (normalized to the glyceraldehyde-3-phosphate dehydrogenase and β-actin expression levels as the most accurate endogenous control gene) of (**A**) heat shock protein 70 (*HSP70*) and (**B**) 8-oxyguanine DNA glycosylase (*OGG-1*) in the jejunum tissue of chickens. The chickens were fed a control diet (CON), CON supplemented with 30 g of hemp extract/kg diet (CBD), CON diet and subjected to *E. coli* LPS and *C. perfringens* challenge (LPS and *C. perfringens*), and CON diet supplemented with 30 g of hemp extract/kg diet and subjected to *E. coli* LPS and *C. perfringens* (CBD + LPS and CBD + *C. perfringens*). Significant differences are indicated by different letters (*p* < 0.05). The error bars indicate the pooled standard deviations in each dietary treatment group for the eight chickens.

The mRNA levels of *HSP70* and *OGG-1* were significantly increased in the *C. perfringens* group compared to the group infected with CBD addition and in the CBD group compared to the CON group (*p* = 0.006). No significant differences were found in the expression of the two genes between the LPS-infected groups with and without CBD addition and in the CON group compared to the CBD + *C. perfringens* group (Figure 4).

#### 2.1.5. The Relative mRNA Expression Levels of Genes Involved in Host Immune Response

**Figure 5:**
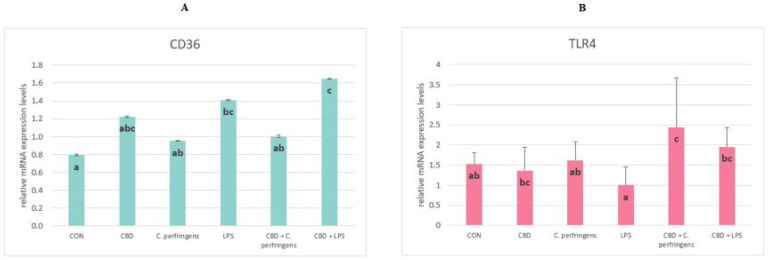
The relative mRNA expression levels (normalized to the glyceraldehyde-3-phosphate dehydrogenase and β-actin expression levels as the most accurate endogenous control gene) of (**A**) CD36 molecule (*CD36*) and (**B**) toll-like receptor 4 (*TLR4*) in the jejunum tissue of chickens. The chickens were fed a control diet (CON), CON supplemented with 30 g of hemp extract/kg diet (CBD), CON diet and subjected to *E. coli* LPS and *C. perfringens* challenge (LPS and *C. perfringens*), and CON diet supplemented with 30 g of hemp extract/kg diet and subjected to *E. coli* LPS and *C. perfringens* (CBD + LPS and CBD + *C. perfringens*). Significant differences are indicated by different letters (*p* < 0.05). The error bars indicate the pooled standard deviations in each dietary treatment group for the eight chickens.

The expression level of *CD36* was significantly increased in the LPS-infected group with CBD supplementation compared to all other study groups (*p* = 0.026) aside from the LPS and CBD groups. There were no significant differences between the group infected with *C. perfringens* and those with and without CBD addition. The *TLR4* levels were significantly increased in the infected groups with CBD supplementation compared to the infected control groups and in the group with CBD supplementation than in the LPS-infected group without supplementation (*p* = 0.002). There were no significant differences among the control group and the *C. perfringens*-infected group (Figure 5).

The expression levels of the *P53*, *ZO-1*, *ZO-2*, *OCLN*, *MUC-5B*, *MUC-2*, *E-cad*, *GPX-1*, *CLDN-1*, *Tac1*, *CNR1* and *CNR2* genes did not differ between the dietary treatment groups (*p* > 0.05).

### 2.2. Correlations Between mRNA Expression Levels of PPARs and Selected Genes

The results of the correlations are presented in Table 1. There was a statistically significant positive correlation between *PPARγ* and *ZO-2*, *E-cad* and *CLDN-1* (*p* = 0.001), *CD36* (*p* = 0.002), *OCLN* (*p* = 0.015), *CLDN-3* and *JAM-2* (*p* = 0.022 and 0.026, respectively), *ZO-1* (*p* = 0.052); between *PPARα* and *OCLN* and *CNR1* (*p* = 0.001), *E-cad* (*p* = 0.003), *MUC-5B* and *ZO-2* (*p* = 0.005 and 0.007, respectively), *CLDN-1* and *CLDN-3* (*p* = 0.014 and 0.017, respectively), *TLR4* (*p* = 0.027), *ZO-1* and *MUC-2* (*p* = 0.030 and 0.032, respectively); between *PPARδ* and *ZO-2* and *MUC-2* (*p* = 0.001), *ZO-1* and *JAM-2* (*p* = 0.030 and 0.033, respectively), and *OCLN* (*p* = 0.051).

### 2.3. Effect of CBD on FITC-d Concentration in Blood of Challenged and Non-Challenged Birds

**Figure 6:**
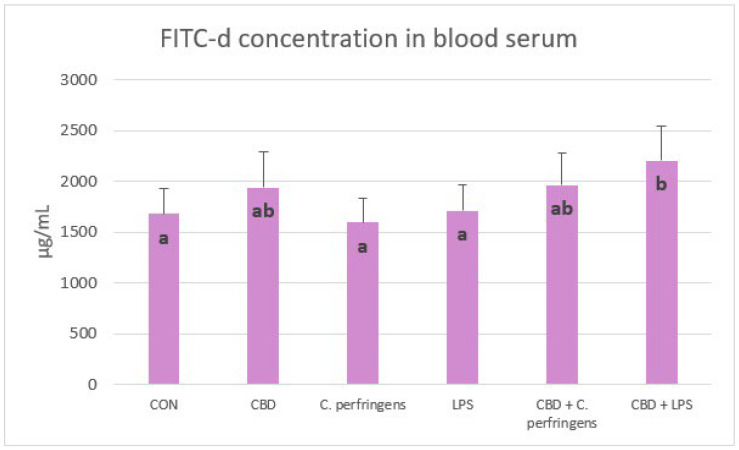
Serum fluorescein isothiocyanate-dextran (FITC-d) concentrations (μg/mL) in chickens fed control diets or CBD supplemented diet and as a result of *E. coli* LPS and *C. perfringens* challenge or no challenge. Significant differences are indicated by different letters (*p* < 0.05). The error bars indicate the pooled standard deviations in each dietary treatment group for the eight chickens. The SEM is 49.45.

Serum FITC-d concentrations were significantly lower in control and infected groups than in birds infected with *E. coli* LPS fed a diet with CBD (*p* = 0.001). In contrast, FITC-d levels were not significantly different between the control and infected groups without CBD supplementation and between CBD, CBD + *C. perfringens* and CBD + LPS groups (Figure 6).

### 2.4. Correlations Between Gene Expression and FITC-d Concentration

The results of correlations are shown in Table 2. There was a statistically significant positive correlation between FITC-d, and *TLR4* (*p* = 0.036), *CLDN* (*p* = 0.038), *CD36* (*p* = 0.040), and a negative correlation between FITC-d, and *IAP* and *PPARα* (*p* = 0.032).

### 2.5. The Effect of CBD on Cortisol and Endotoxin Concentration in the Blood

**Figure 7:**
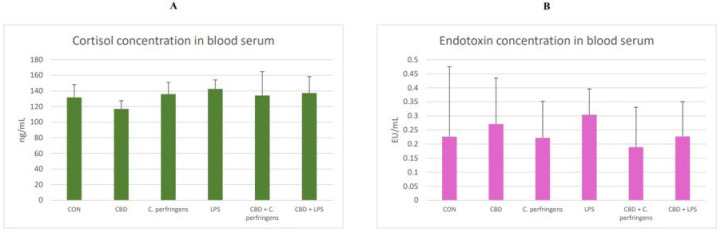
(**A**) Cortisol (ng/mL) and (**B**) endotoxin (LPS, EU/mL (endotoxin units)) concentrations in the blood serum of the chickens. The chickens were fed a control diet (CON), CON supplemented with 30 g of hemp extract/kg diet (CBD), CON diet and subjected to *E. coli* LPS and *C. perfringens* challenge (LPS and *C. perfringens*), CON diet supplemented with 30 g of hemp extract/kg diet and subjected to *E. coli* LPS and *C. perfringens* (CBD + LPS and CBD + *C. perfringens*). The error bars indicate the pooled standard deviations in each dietary treatment group for the eight chickens. The SEM for cortisol is 2.77, and for endotoxin, it is 0.023.

The results of the cortisol and endotoxin (LPS) concentration in the blood serum of challenged and non-challenged chickens are presented in Figure 7. The concentrations of both did not differ between the dietary treatment groups (*p* > 0.05).

### 2.6. Correlations Between Gene Expression and Cortisol Concentration

The correlation results are shown in Table 3. There was a significant negative correlation between the cortisol concentration and *CNR1* (*p* = 0.037) and *JAM-2* (*p* = 0.047).

### 2.7. Correlations Between Gene Expression and Endotoxin Concentration

Table 4 presents the results of the correlations. There was a statistically significant negative correlation between the endotoxin concentration and *CLDN-1* (*p* = 0.022) and *CD36* (*p* = 0.028).

## 3. Discussion

The *PPAR* family regulates the transcription of genes involved in cellular differentiation, lipoprotein, and lipid metabolism, influencing cell proliferation, glucose and energy homeostasis, and the inflammatory and immune response in different tissues and cells [42]. In the present experiment, our focus was directed at the interactions between *PPAR*s and the gastrointestinal response to various challenge agents. Although studies by other authors [43] indicate that *PPAR* gene expression is higher in the kidney or liver than in the intestinal mucosa, it was hypothesized that their expression in the intestinal mucosa may also play a crucial role in the initiation of the gastrointestinal-related immune response due to the role of lipids in regulating this response [44]. Lipids have been shown to be important for the optimal inclusion and synthesis of long-chain polyunsaturated fatty acids in the membrane phospholipids of immune cells, also associated with the gut immune system [45]. The current results showed that both CBD and the challenge had no significant effect on the expression levels of the *PPARδ* and *PPARγ* genes in the small intestine, but significantly affected the expression level of the *PPARα* gene. Perhaps the lack of significant impacts on *PPARγ* is due to the fact that *PPARγ* induction in the small intestine is directly related to epithelial cell differentiation [46] or to the animal species or section of the gastrointestinal tract, as De Filippis et al. [47] showed that the anti-inflammatory effects of CBD are *PPARγ*-mediated in the gastrointestinal system in mice treated with LPS. The present results may be attributed to the role of *PPARα* in regulating the rate of glucose and fatty acid oxidation. This could be because *PPARα* expression corresponds to the area of the gastrointestinal tract where most lipids are absorbed, potentially interacting with the immune response [48]. The current results show a significant reduction in the *PPARα* gene expression levels in both challenged groups, confirming the strong dependence of the stress factor interaction on the *PPARα* expression levels [49]. Interestingly, in the group challenged with *C. perfringens* but not with LPS and concomitantly supplemented with CBD, the *PPARα* expression levels increased significantly, indicating that the mechanism of interaction between CBD and *PPARα* is different depending on the stress factor. These results are partially in line with that of Gharib-Naseri et al. [50], who showed that *C. perfringens* infection affected the gut fatty acid metabolism and absorption in chickens, modulating the expression of the genes, their respective pathways, and their functions, which may differ depending on the challenge conditions. In addition, evidence suggests that *PPARα* may counteract inflammation through multiple distinct mechanisms and affect acute and chronic inflammatory processes [51]. This effect can be enhanced by CBD, as indicated by the results presented herein.

The claudin family (*CLDN* and *CLDN-3*) is important in tight junction formation and its function. They are major constituents of the tight junction complexes that regulate the integrity and permeability of epithelia, serving as a physical barrier to prevent water and solutes from passing freely through the paracellular space and forming continuous seals around cells. In addition, claudins are also a low-affinity receptor for *Clostridium perfringens* enterotoxin [52]. *JAM-2* belongs to the junctional adhesion molecule (JAM) family. Type I membrane protein, which is encoded by this gene, acts as an adhesive ligand for interacting with a variety of immune cell types and is located at the tight junctions of both epithelial and endothelial cells [53]. However, in the current study, supplementation with CBD (alone with no challenge) did not improve the expression level of *CLDN*. However, the CBD supplementation improved the *CLDN* expression level in the challenged groups. Moreover, a clear decline in the *CLDN-3* expression level was observed in all of the challenged but not CBD-supplemented groups compared to the other groups, indicating that CBD can have a modulatory effect on the level of *CLDN-3* expression. The same was true for the *JAM-2* expression level in the LPS-challenged but not *C. perfringens*-challenged groups. In the present experiment, changes in the expression levels of the selected TJP genes varied due to the challenge factor used. Compared to the positive controls, the addition of CBD to the diets caused a significant increase in the *CLDN* expression levels, decreased the *JAM-2* expression levels, and did not significantly affect the *CLDN-3* expression levels. In contrast, only an increase in the *JAM-2* expression levels was found for LPS. This indicates that CBD only mediates specific mechanisms of response to the stress factor. *C. perfringens* bacteria secrete toxins and may weaken the TJP barrier in the intestines, leading to malabsorption. Awad et al. [54] summarized that *C. perfringens* produce endotoxin, which disrupts intestinal mucosal barrier function and increases intercellular permeability in chickens. Since the selected TJPs including claudin proteins play an important role in the regulation of cell signaling, it may be possible that changes in the expression of TJP genes in the current study were more pronounced than in the case of LPS. This might also indicate that CBD may be involved in the regulation of cell signaling during inflammation.

The functions of *GLP-2* include protective signaling during an inflammatory state, stimulating intestinal growth, and increasing the villus height in the small intestine. Intestinal alkaline phosphatase promotes colonization of the intestine with commensal organisms as well as plays a role in gut mucosal defense and the inactivation of pathogens [55]. In the present study, it was found that in the case of the *GLP-2* expression level, the supplementation of CBD significantly decreased the expression level of this gene in the intestine of birds infected with *C. perfringens*, indicating that CBD ameliorated the inflammation caused by this pathogen. The same relationship was found for the response of birds to LPS and for the *IAP* expression levels. It is also important to note that the expression levels of both genes in the intestine in the positive control groups were at the same level as that in the challenged + CBD supplementation groups, indicating that the addition of CBD alone to the birds’ diets did not cause a stressful effect manifested by the activation of a defense mechanism involving *GLP-2* and *IAP*. Cani et al. [56] demonstrated, in a probiotic-treated mouse model, lower LPS levels in plasma and reduced expression of oxidative and inflammatory cells in the liver. This, in turn, reduced the intestinal permeability through the increased expression levels of TJPs. The mice showed an increased endogenous production of *GLP-2* during obesity-induced inflammation. Similarly, in the current experiment, the *GLP-2* expression levels increased in chickens during inflammation induced by *C. perfringens* or *E. coli* LPS. In contrast, chickens challenged with dexamethasone stress showed a significant increase in the *IAP* expression levels, indicating its active involvement in the gastrointestinal-related immune response [57]. The reason for the inverse reaction in the case of the *IAP* gene expression levels in the avian gut to the stress agent may be due to the severity of the challenge, as in the current research model (for both *C. perfringens* and *E. coli* LPS), the challenge applied was mild.

On the other hand, *HSP70* and *OGG-1* are genes involved in protecting cells from oxidative stress. Heat shock protein 70 is induced in response to cell stress, protects cells from injury, and promotes the refolding of denatured proteins. *OGG-1* regulates the transcription of various oxidative stress-response genes, prevents the accumulation of mutations, and has an integral role in maintaining cellular homeostasis under oxidative stress. Although both genes are associated with regulation of the gastrointestinal response to pathogen-induced oxidative stress in the current experiment, differential activity was observed, depending on the stress factor. In the case of *HSP70*, which promotes the protection of cells from the lethal effects of oxidative stress [58], a significant reduction was found in its expression levels in *C. perfringens*-challenged birds given CBD compared to the challenged-only birds, while no differences were observed for the LPS-challenged (LPS-challenged vs. LPS-challenged and CBD-supplemented) birds. This indicates that CBD supports mechanisms protecting intestinal cells from lethal effects. In another study, the expression level of *HSP70* in chickens subjected to acute heat stress was found, where this gene plays an important role in the response to oxidative stress [59]. That study demonstrated a strong positive correlation between digestive enzyme activity and *HSP70* expression levels under heat stress, proving that *HSP70* may improve gut function during acute heat stress. With regard to the current results, it can be concluded that CBD reduced the oxidative stress associated with *C. perfringens* infection because the expression level of *HSP70* was the same in the challenged chickens as in the control group. In contrast, the fact that these levels did not differ from the CBD group indicates that the activity of CBD in regulating oxidative stress is induced by a specific stress factor. The lack of significant response in the *OGG-1* gene expression level is unclear. However, in the turkey model challenged with either *C. perfringens* or with *E. coli* LPS, no significant response was found in the jejunum to this gene expression level [60]. Because *OGG-1* recognizes modified bases of DNA and initiates the repair process of DNA strands at the site of damage [61], it may indicate that neither *C. perfringens* nor *E. coli* LPS harmed the mucosal cell DNA integrity in the current study.

*CD36* and *TLR4* receptors are involved in processes of innate immunity. The functions of *CD36* are related to the transport of fatty acids into the cell for the lipid synthesis of their metabolism, the uptake of cholesterol, and consequently, regulation of the inflammatory response to inflammation. Whereas *TLR4* is a primary signal of the innate immune response pathway, which plays a key role in the defense mechanism against infectious diseases, evidence suggests that endotoxin is also recognized via *TLR4* receptors [62,63]. In the current study, the *CD36* activity did not change due to challenge vs. CBD supplementation, although a significant response was found regarding *TLR4*. CBD supplementation in both cases increased the expression level of the *TLR4* gene. This response is consistent with other reports in which an upregulation of this gene expression has been reported in the case of challenge factors in the birds’ immune tissues [64]. In the mentioned study, the expression level of *TLR4* was upregulated in the spleen (although there was no significant effect in the ileum) on day 1 post-challenge with *C. perfringens*, and then it dropped to the base level. This may partially explain the observed response, in which the post-challenge expression level of *TLR4* in the challenged birds did not differ from the CON. The current results also indicate a positive effect of CBD in this regard, since in both challenged groups, except for the CBD-supplemented group, this expression was upregulated at this time point. This action is beneficial for the host, since the recognition of potential pathogens by the innate immune system is the function of PRRs, which include the toll-like receptors (including *TLR4*).

The *PPAR* family plays a regulatory role in the host response to different pathogenic stimuli. Although many studies have been conducted on the interaction between the host and *PPAR*s, new pathways for their activity in birds are still being discovered [65], and little is known about the potential role of *PPARδ* activation by cannabinoids and the effects of phytocannabinoids on *PPARα* [6]. The present experiment verified the regulatory properties of *PPAR*s on the functional status of the gastrointestinal tract. The study revealed a significant correlation between determinants of intestinal barrier integrity in chickens maintained under optimal and induced stress conditions. Based on the correlation between *PPAR*s and the gene expression levels, a significant correlation was found between *PPARγ* vs. eight genes (*ZO-1*, *ZO-2*, *OCLN*, *JAM-2*, *E-cad*, *CLDN-3*, *CLDN-1*, *CD36*), *PPARα* vs. ten genes (*ZO-1*, *ZO-2*, *TLR4*, *OCLN*, *MUC-5B*, *MUC-2*, *E-cad*, *CLDN-3*, *CLDN-1*, *CD36*), and *PPARδ* vs. five genes (*ZO-1*, *ZO-2*, *OCLN*, *MUC-2*, *JAM-2*), which determine the formation of TJPs, immune response, and oxidative stress response in chickens. Particularly noteworthy is that in all cases in which a significant correlation was found, it was positive, and the most significant positive correlation between the *PPAR*s studied was between genes responsible for TJPs including *ZO-1*, *ZO-2*, and *OCLN*. The current study may also point to a specific mechanism for supporting the immune system of the host by enhancing the integrity of the gut barrier through *PPAR*s. Although the host response varied depending on the challenge factor (*C. perfringens* vs. LPS), globally, the response mechanism proceeded by increasing the expression level of the selected genes encoding TJPs. The results may be significant because other studies have shown that although *PPAR* expression in birds manifests in different tissues of the biological system, its expression rate varies to a high extent depending on the tissue [66]. In light of the above, the current results seem to show a constant association in the jejunum mucosa of chickens. Similarly, other researchers have also revealed a key role of *PPAR*s in supporting gut function, particularly in challenging conditions due to the effects on the expression of TJPs including *ZO-1*, *ZO-2*, mucins, claudins, or occludins, or may even manifest a neuroprotective effect [67,68]. Although there have been a few studies investigating the association between *PPAR*s and TJPs in poultry, the current results on a chicken model are in line with other reports indicating that this association might be highly conservative. It is also interesting to note that the current study found a more pronounced response of gene expression in the challenged birds when CBD was supplemented. According to O’Sullivan [6], CBD activates the different isoforms of *PPAR*s, and this, in turn, mediates anti-inflammatory actions. This may explain why different host responses to challenge stimuli in CBD-supplemented birds have mostly been observed compared to the challenged birds alone.

In the present study, a FITC-d test was performed to verify whether dietary treatments affect gut permeability in birds. This test can be widely applied to study the gut response to challenge factors as it is simply based on the difference between the concentration of 4-kDa fluorescein isothiocyanate-dextran in the blood as a response to gavage, which is measured in a defined time period. The results indicate that the higher the concentration of FITC-d in the blood, the higher the permeability of the gut manifested by the host. In the present study, there is no logical explanation as to why the permeability of the gut did not increase due to the challenge of either *C. perfringens* or LPS from *E. coli*. It is possible that the challenge model was not severe enough as it did not prevent molecules of such a mass from passing the gut barrier. Another explanation is that the response to FITC-d transposing in chickens varies for different reasons (i.e., challenge conditions, type of diet, age of birds, etc.). However, most studies have shown an increase in the blood concentration of FITC-d due to different challenges [69]. There have also been reports indicating no response due to challenges [70,71] compared to the control group. Regarding the increased level of FITC-d in birds exposed to LPS and fed CBD when compared to the LPS-challenged group alone, as reported in the current study, this might be due to the potential properties of CBD in gut collagen degradation through the increased activity of collagenase in the gut because this response was found in a previous study by the authors [27]. This may also partially explain the positive correlation found for the expression levels of the *TLR4*, *CD36*, and *CLDN* genes in the jejunum and blood FITC-d. In addition, the negative correlation between FITC-d and *PPARα* may confirm the study by Mazzon et al. [72], which provides evidence that the degree of TJ permeability in the mouse model associated with experimental colitis is modulated by the *PPARα* pathway.

In the present study, cortisol measurements were applied to investigate the host’s response to treatments. Cortisol concentration is a commonly used indicator to assess acute stress [73]. Despite ample previous evidence that corticosterone is the main glucocorticoid produced by the adrenal glands of birds [74,75], the authors of the present study decided to investigate the serum cortisol levels in chickens. There are several factors that limit the reliability of using corticosterone in studies of stress including heat stress in poultry. First, it is possible to reduce the corticosterone levels to baseline levels through a negative feedback mechanism [76]. In addition, corticosterone levels fluctuate with diurnal rhythm and reproductive cycle [76,77]. A recent study by Kim et al. [78] found cortisol levels above 10 ng/mL, although its concentration in chickens is thought to only be at very low levels. Li et al. [79] studied the effect of electrical stunning before slaughter on the serum cortisol levels as an indicator of stress. Their study showed that the cortisol levels were almost twice as high in the non-stunned group. Research by Gou et al. [73] also showed statistically significant changes in the cortisol levels in birds. Tetel et al. [80] concluded that in addition to corticosterone, cortisol is also stimuli-responsive and should be studied further in poultry. These studies show that cortisol levels should also be considered in poultry studies as a marker of stress. In the present experiment, it was found that there was no significant influence of dietary intervention on the concentration of cortisol in the blood of birds. However, it was found that there was a negative correlation between the gene expression levels in the jejunum including *JAM-2* and *CNR1* and the blood concentration of cortisol. The first gene is associated with the formation of TJPs, whereas the second is the cannabinoid receptor-coding gene. In the first case, the reason is that TJPs are being disrupted, which increases patterns of stress, whereas the second phenomenon seems to be associated with CBD action. This may also partially explain the different responses of challenged birds supplemented vs. not supplemented with CBD, which could have been associated with the activation of the CBD-1 receptor.

In the present experiment, neither the CBD treatment nor the provocation used had any effect on the endotoxin concentration in the blood of the chickens. However, it was found that there was a negative correlation between the endotoxin concentration in the blood and the expression level of genes in the jejunum such as *CLDN-1* and *CD36*. This response is consistent with the gene expression described in the first section. The disruption of intestinal mucosal function in the present study resulted in higher endotoxin concentrations in the blood. However, this response was rather small, as only two of all genes tested were significantly associated with endotoxin.

## 4. Materials and Methods

### 4.1. Chicken Experiment, Diets, and Applied Experimental Challenges

#### 4.1.1. Cannabis Extract Chemical Composition

Hemp panicles (*Cannabis sativa*) were obtained from plants collected at the Institute of Natural Fibers and Medical Plants in Poznan, Poland in 2019. Plants were cultivated from certified seeds in compliance with institutional, national, and international regulations. The supercritical carbon dioxide extract of hemp was obtained from the Supercritical Extraction Plant of Institute of New Chemical Synthesis, Puławy, Poland. After evaporation, the hemp extract contained 12% CBD, 0.38% tetrahydrocannabinolic acid, and 0.49% tetrahydrocannabinol, as determined by HPLC [81]. Therefore, considering the inclusion level (30 g/kg diet) of the CBD extract in the diet, the final concentrations were 3.6 g CBD per 1 kg feed (0.36%) and 0.147 g tetrahydrocannabinol per 1 kg of feed (0.015%) [81].

#### 4.1.2. Chicken Experiment and Diets

Approval for the study (Resolution No. 54/2019 of 30 July 2019) was obtained from the Local Ethics Committee for animal testing at UWM Olsztyn, Poland. All procedures involving animals were performed in accordance with the Polish Law on Animal Protection, Polish Law for the Animal Care and Use, EU regulations (Directive 2010/63/EU), and the Code of Ethics of the World Medical Association (Declaration of Helsinki). The present experiment also complied with the ARRIVE guidelines.

**Figure 8:**
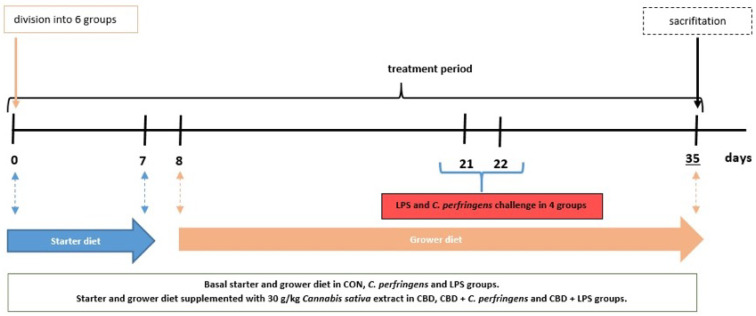
Study design. Blue arrow represents feeding period of chickens on starter diet (0–7 days); orange arrow represents feeding period of chickens on grower diet (8–35 days).

On the day of hatching, a total of 204 Ross 308 male broilers were purchased from a local hatchery. Upon arrival at the experimental unit, the birds were divided into six treatment groups according to average body weight, each containing 34 chicks. Chickens received a starter diet on days 0–7, and a grower diet from day 8 until the end of the experiment. The birds consumed diets similar to a commercial one formulated to meet or exceed the Ross 308 broilers nutritional requirements according to their age. Throughout the experiment, access to drinking water and feed was unrestricted. The birds in each group were kept on bedding in pens, and housing conditions, such as light cycle (an 18-h day cycle and a 6-h night cycle), humidity, and temperature, were maintained according to standard management practices for commercial poultry houses. Throughout the experiment, broilers in the control (CON) group were fed a basal diet, while those in the CBD group consumed a control diet supplemented with 30 g/kg of *C. sativa* extract. Chickens in the LPS and *C. perfringens* (positive control) groups were challenged with *E. coli* LPS and *C. perfringens*, respectively, and received the CON diet. Birds in the CBD + *E. coli* LPS and CBD + *C. perfringens* groups consumed the same diet as the CBD group and were challenged in addition. The division into groups is shown in Table 5. Using a CL-2 CPM (CPM, Colcord, OK, USA) laboratory pellet mill, the diets were cold pelleted. A simple diagram of the study is shown in Figure 8.

#### 4.1.3. Applied Experimental Challenges and Sampling Procedure

*C. perfringens* or LPS from *E. coli* in the respective challenged groups were given to the birds at 21 and 22 days of age. After 4 h of food deprivation, the birds from LPS and CBD + LPS were weighed and orally administered (*per os*) LPS (*Escherichia coli* O55:B5 serotype, Sigma Chemical, St. Louis, MO, USA) in 0.9% NaCl (0.5 mg/mL) at a dose of 1 mL, which contained 250 μg/kg body weight of LPS [81]. On the same days, the birds in the CBD + *C. perfringens* and *C. perfringens* groups were given (*per os*) 1 mL inoculum (brain heart infusion medium) containing approximately 10^8^ CFU/mL *C. perfringens* strain 56 type A bacteria [81], which was isolated from infected chickens and a coccidial cocktail to create a favorable environment for C. *perfringens* proliferation [27]. According to the supplier declaration, the strain was previously confirmed to be β-toxin- and enterotoxin-negative, and α-toxin- and NetB toxin-positive (Ghent University, Merelbeke, Belgium). The birds in the CBD and CON groups were each administered 1 mL of sterile brain heart infusion medium with a coccidial cocktail and sterile saline as a placebo for the *C. perfringens* or LPS-challenged groups.

At day 35, the birds were weighed, and eight broilers from each group were electrically stunned (150 mA, 350 Hz) and decapitated. Blood was drawn from the wing vein into serum tubes and centrifuged after 30 min. Subsequently, the entire digestive tract was removed from the same birds, and a section of the small intestine (at Meckel’s diverticulum) was collected. The samples of jejunum and serum were immediately frozen at −80 °C for ELISA and Real-Time PCR analysis.

### 4.2. Real-Time PCR

Using the Total RNA Mini Kit (A&A Biotechnology, Gdynia, Poland; Cat. No.: 031-100), the total mRNA was isolated from small intestine samples according to the provided protocol. The yield of isolated RNA was assessed spectrophotometrically (Nanodrop, NanoDrop Technologies, Wilmington, DE, USA). Integrity was evaluated electrophoretically by separation on a 1.5% agarose gel containing ethidium bromide. To synthesize complementary DNA (cDNA), 1000 ng/mL mRNA from jejunum tissue in a total volume of 20 μL was reverse-transcribed using the Maxima First Strand cDNA Synthesis Kit for RT-qPCR with dsDNase (ThermoFisher Scientific, Warsaw, Poland; Cat. No.: K1672) as indicated by the manufacturer’s instructions. The chicken (*Gallus gallus*) specific primers used to determine the test and housekeeping gene expression were designed using Primer designing tool NCBI software (National Library of Medicine, Bethesda, MD, USA; https://www.ncbi.nlm.nih.gov/tools/primer-blast/) and synthesized by Genomed (Warsaw, Poland) including heat shock protein 70 (*HSP70*), trefoil factor 2 (*TFF2*), peroxisome proliferator-activated receptor alpha (*PPARα*), peroxisome proliferator-activated receptor gamma (*PPARγ*), peroxisome proliferator-activated receptor delta (*PPARδ*), tumor protein 53 (*P53*), zonula occludens 2 (*ZO-2*), zonula occludens 1 (*ZO-1*), toll-like receptor 4 (*TLR4*), occludin (*OCLD*), mucin 2 (*MUC-2*), mucin 5B (*MUC-5B*), junctional adhesion molecule 2 (*JAM-2*), intestinal alkaline phosphatase (*IAP*), e-cadherin (*E-cad*), claudin-3 (*CLDN-3*), glucagon-like peptide 2 (*GLP-2*), 8-oxyguanine DNA glycosylase (*OGG-1*), glutathione peroxidase 1 (*GPX-1*), claudin 1 (*CLDN-1*), CD36 molecule (*CD36*), claudin (*CLDN*), tachykinin precursor 1 (*Tac1*), cannabinoid receptor 1 (*CNR1*), and cannabinoid receptor 2 (*CNR2*). Real-time qPCR was carried using 2 × AMPLIFYME SG No-Rox Mix (Blirt, Gdańsk, Poland; Cat. No.: AM01-020) in a total volume of 15 μL containing 1.5 μL cDNA template, 2 × 0.5 μL primers (0.5 mM), 5 μL RNAse-free H_2_O, and 7.5 μL Master Mix. Amplification was performed using a Rotor Gene 6000 thermocycler (Corbett Research, Mortlake, Australia) according to the following PCR protocol: enzyme activation (one cycle at 95 °C for 3 min), denaturation (40 cycles at 95 °C for 5 s), annealing (60 °C for 10 s), and elongation (72 °C for 5 s), followed by product stabilization (one cycle at 72 °C for 7 min). The melting curve was performed in 0.5 °C intervals at 70–95 °C. Each reaction included negative controls without the cDNA template. For each cDNA sample, the real-time qPCR reaction was performed twice in duplicate. The identity of the PCR products was confirmed by direct sequencing. Relative gene expression was calculated using the comparative quantification option of Rotor Gene 6000 1.7 software (Qiagen GmbH, Hilden, Germany) and determined using the Relative Expression Software Tool, http://rest.gene-quantification.info/, based on the PCR efficiency correction algorithm. β-Actin (ACTB), glyceraldehyde-3-phosphate dehydrogenase (GADPH), phosphoglycerate kinase 1 (PGK1), ribosomal protein L12 (RPL12), and histone deacetylase (HDAC) genes were tested as host genes using NormFinder software (MOMA, Aarhus N, Denmark; https://www.moma.dk/software/normfinder). GAPDH and ACTB genes were used as endogenous controls for normalizing gene expression. The results were presented as the relative expression of the housekeeping gene vs. target gene and relative gene expression for a selected group of chickens. The primer sequences are listed in Table 6.

### 4.3. Determination of the FITC-D Concentration in Blood Serum

To test the intestinal permeability, a total of eight 35-day-old chickens in each group were orally administered 1 mL aqueous solution of fluorescein isothiocyanate-dextran (FITC-D; Sigma Aldrich, St. Louis, MO, USA; Cat. No.: FD4) at a dose of 2.2 mg/bird according to a previous report [82]. Two chickens from each group received saline as a control serum. Two and a half hours after being administered FITC-D, the broilers were sacrificed. Blood samples were taken from the wing vein into a blood collection tube for serum (BD Vacutainer, Franklin Lakes, NJ, USA) and centrifuged (15 min, 3000× *g*) [66]. The separated serum was then aliquoted and shorted in amber tubes at −80 °C. Serum from the control broilers was used to prepare a standard curve for FITC-D. All samples from the non-FITC-d broilers were diluted in sterile saline at a ratio of 1:5. All dilutions and standard curves were performed in a microtiter dilution plate. The final volume of the samples was 100 uL/well in the plate reader. Each 96-well assay plate run with samples contained its standard curve. Diluted samples were plated in duplicate. Fluorescence was measured at 528 nm emission and 485 nm excitation using a spectrophotometer with a microplate reader (Multiskan Sky, Thermo Scientific, Rockford, IL, USA). Based on a calculated standard curve, the levels of fluorescence in the samples were converted to respective FITC-d micrograms per milliliter of serum.

### 4.4. Determination of Cortisol in Blood Serum

The cortisol level in blood was determined using a commercial ELISA kit for chickens (MyBioSource, Eersel, The Netherlands; Cat. No.: MBS265227). To obtain the serum, whole blood was centrifuged for 10 min at 2500 rpm. The separated serum was then divided into 0.5 mL portions and shorted at −20 °C. All remaining steps of the ELISA were completed following the manufacturer’s instructions. Standards and samples were placed in duplicate on the plate. Absorbance was measured at 450 nm using a spectrophotometer with a microplate reader (Multiskan Sky, Thermo Scientific, Rockford, IL, USA). Based on a calculated standard curve (with a detection range of 200 ng/mL), the levels of absorbance in the samples were converted to the respective cortisol nanograms per milliliter of serum.

### 4.5. Determination of Endotoxin in Blood Serum

Gram-negative bacterial endotoxin levels in blood samples were determined using the Pierce LAL Chromogenic Endotoxin Quantitation Kit (Thermo Scientific, Rockford, IL, USA; Cat. No.: 88282). The blood was taken from a wing vein and centrifuged for 10 min at 2000 rpm to obtain the serum. The separated serum was then aliquoted and stored at −20 °C. The analysis was performed according the provided protocol. Standards and samples were placed in duplicate on the plate. Absorbance was measured at 410 nm using a spectrophotometer with a microplate reader (Multiskan Sky, Thermo Scientific, Rockford, IL, USA). A standard curve (linear regression, with a range of 1.25 EU/mL) was then prepared and used to determine the endotoxin concentration in each unknown sample.

### 4.6. Statistics

All data were expressed as the means of eight birds per group. The variability was presented as the pooled standard deviation (SD) values or pooled standard error of the mean (SEM) test. The Shapiro–Wilk and Levene tests were applied to test the model assumptions of normality and homogeneity of variance. Differences among groups were estimated using one-way ANOVA with the least significant difference (LSD) test. Differences resulting in *p* <  0.05 were considered statistically significant. Correlations between the concentration of cortisol, endotoxin, FITC-d, and gene expression were evaluated with a Pearson correlation analysis. Statistical calculations were performed using STATGRAPHICS Centurion XVI ver. 16.1.03 software.

## 5. Conclusions

The present research provides strong evidence that there is a close association between *PPAR*s and the gut response of chickens to different stress factors. This association mostly manifested in modulating the expression level of the jejunum genes, influencing the formation of TJPs, immune response, and oxidative stress response. However, the most constant association was between *PPARα*, *PPARβ*, *PPARδ*, and genes encoding TJPs including *ZO-1*, *ZO-2*, and *OCLN*, which determine the gut barrier integrity. Expression of the investigated PPARs in the jejunum was more pronounced in the challenge conditions but varied depending on the challenge factor (*C. perfringens* vs. *E. coli* LPS). Dietary supplementation of CBD actively mediated the expression rate of PPARs, but the interaction mechanism between CBD and *PPAR*s differed depending on the stress conditions used. CBD did not reduce the intestinal permeability under induced infection. Moreover, it did not cause stress, as indicated by the levels of genes involved in oxidative stress and the absence of elevated blood cortisol and endotoxin levels. In addition, CBD exhibited a supportive effect on mechanisms to protect the intestinal cells from lethal effects. The current results seem to show a constant *PPAR* association with the jejunum mucosa of chickens. The present study indicates the importance of research toward understanding the action of PPAR mechanisms as a target to enhance intestinal barrier function in chickens. In addition, the present study provided new results on the biological action and mechanism of CBD in chickens.

It should be noted that the study presented herein is directed at the poultry industry. Chicken are not a suitable model for translational medicine or the transfer of results to other species such as humans or companion animals. It should also be kept in mind that as we aimed to induce a subclinical, not acute, form of the infection, which, as mentioned, does not show typical clinical signs, it was not straightforward to fully assess the changes induced by infectious agents throughout the experiment. Additionally, cannabidiol is currently not authorized for use as an animal feed additive in EU member states. The European Food Safety Authority (EFSA) must approve of all the feed supplements. Cannabidiol is also regulated under the novel food regulations. Therefore, future research, such as this, which shows the high potential of CBD, is very much required to bring cannabidiol into legal use.

## Figures and Tables

**Table 1:** Correlations between gene expression in the jejunum.

Gene	*PPARγ*		*PPARα*		*PPARδ*	
	*r*	*p*-Value	*r*	*p*-Value	*r*	*p*-Value
*HSP70*	0.083	0.598	−0.171	0.262	0.134	0.387
*TFF2*	0.109	0.477	−0.015	0.092	0.064	0.672
*P53*	0.22	0.152	0.247	0.097	0.228	0.132
*ZO-2*	0.559	0.001 *	0.411	0.007 *	0.657	0.001 *
*ZO-1*	0.295	0.052 *	0.320	0.030 *	0.324	0.030 *
*TLR4*	0.044	0.776	0.323	0.027 *	−0.02	0.896
*OCLN*	0.361	0.015 *	0.459	0.001 *	0.290	0.051 *
*MUC-2*	0.226	0.136	0.313	0.032 *	0.474	0.001 *
*MUC-5B*	0.194	0.203	0.404	0.005 *	0.056	0.71
*JAM-2*	0.339	0.026 *	0.220	0.152	0.325	0.033 *
*IAP*	0.069	0.651	−0.085	0.567	0.014	0.923
*E-cad*	0.545	0.001 *	0.436	0.003 *	0.24	0.113
*CLDN-3*	0.346	0.022 *	0.351	0.017 *	0.212	0.162
*GLP-2*	0.021	0.89	−0.043	0.755	0.21	0.161
*OGG-1*	0.139	0.376	0.176	0.248	0.236	0.123
*GPX-1*	0.01	0.954	−0.085	0.605	−0.196	0.233
*CLDN-1*	0.622	0.001 *	0.366	0.014 *	0.25	0.102
*CD36*	0.449	0.002 *	0.177	0.299	0.051	0.736
*CLDN*	0.101	0.526	0.209	0.173	0.1	0.518
*CNR1*	0.197	0.194	0.459	0.001 *	0.06	0.687

Calculated Pearson’s correlation coefficient between the mRNA expression levels of PPARs and select genes determining gut integrity, involved in GIT inflammation defense, oxidative stress, and immune response in the jejunum of chickens subjected to different challenges. * Significant correlation at *p* < 0.05. Abbreviations: *HSP70*, heat shock protein 70; *TFF2*, trefoil factor 2; *PPARα*, peroxisome proliferator-activated receptor alpha; *PPARγ*, peroxisome proliferator-activated receptor gamma; *PPARδ*, peroxisome proliferator-activated receptor delta; *P53*, tumor protein 53; *ZO-2*, zonula occludens 2; *ZO-1*, zonula occludens 1; *TLR4*, toll-like receptor 4; *OCLD*, occludin; *MUC-2*, mucin 2; *MUC-5B*, mucin 5B; *JAM-2*, junctional adhesion molecule 2; *IAP*, intestinal alkaline phosphatase; *E-cad*, e-cadherin; *CLDN-3*, claudin-3; *GLP-2*, glucagon like peptide 2; *OGG-1*, 8-oxyguanine DNA glycosylase; *GPX-1*, glutathione peroxidase 1; *CLDN-1*, claudin 1; *CD36*, CD36 molecule; *CLDN*, claudin; *CNR1*, cannabinoid receptor 1.

**Table 2:** Correlations between gene expression in the jejunum and FITC-d concentration in the blood serum.

Gene	FITC-d	
	*r*	*p*-Value
*HSP70*	−0.105	0.500
*TFF2*	0.213	0.155
*PPARα*	−0.275	0.032 *
*PPARγ*	−0.083	0.590
*PPARδ*	0.005	0.974
*P53*	0.002	0.992
*ZO-2*	0.06	0.972
*ZO-1*	0.206	0.175
*TLR4*	0.311	0.036 *
*OCLN*	0.255	0.087
*MUC-2*	0.147	0.330
*MUC-5B*	0.279	0.062
*JAM-2*	−0.150	0.338
*IAP*	−0.313	0.032 *
*E-cad*	−0.055	0.719
*CLDN-3*	0.189	0.215
*GLP-2*	−0.269	0.071
*OGG-1*	−0.027	0.864
*GPX-1*	−0.141	0.398
*CLDN-1*	−0.083	0.591
*CD36*	0.301	0.040 *
*CLDN*	0.318	0.038 *
*CNR1*	0.258	0.083

Calculated Pearson’s correlation coefficient between gene expression in the jejunum and FITC-d in the blood serum of chickens subjected to different challenges. * Significant correlation at *p* < 0.05. Abbreviations: *HSP70*, heat shock protein 70; *TFF2*, trefoil factor 2; *PPARα*, peroxisome proliferator-activated receptor alpha; *PPARγ*, peroxisome proliferator-activated receptor gamma; *PPARδ*, peroxisome proliferator-activated receptor delta; *P53*, tumor protein 53; *ZO-2*, zonula occludens 2; *ZO-1*, zonula occludens 1; *TLR4*, toll-like receptor 4; *OCLD*, occludin; *MUC-2*, mucin 2; *MUC-5B*, mucin 5B; *JAM-2*, junctional adhesion molecule 2; *IAP*, intestinal alkaline phosphatase; *E-cad*, e-cadherin; *CLDN-3*, claudin-3; *GLP-2*, glucagon like peptide 2; *OGG-1*, 8-oxyguanine DNA glycosylase; *GPX-1*, glutathione peroxidase 1; *CLDN-1*, claudin 1; *CD36*, CD36 molecule; *CLDN*, claudin; *CNR1*, cannabinoid receptor 1.

**Table 3:** Correlations between gene expression in the jejunum and cortisol concentration in the blood serum.

Gene	Cortisol	
	*r*	*p*-Value
*HSP70*	−0.067	0.664
*TFF2*	0.104	0.488
*PPARα*	−0.105	0.478
*PPARγ*	−0.154	0.308
*PPARδ*	−0.21	0.157
*P53*	0.186	0.217
*ZO-2*	−0.024	0.881
*ZO-1*	−0.087	0.566
*TLR4*	−0.047	0.754
*OCLN*	−0.085	0.571
*MUC-2*	−0.105	0.481
*MUC-5B*	−0.24	0.104
*JAM-2*	−0.301	0.047 *
*IAP*	−0.192	0.190
*E-cad*	−0.044	0.771
*CLDN-3*	−0.053	0.726
*GLP-2*	−0.146	0.327
*OGG-1*	−0.218	0.151
*GPX-1*	−0.053	0.748
*CLDN-1*	−0.044	0.775
*CD36*	0.1	0.498
*CLDN*	0.186	0.226
*CNR1*	−0.305	0.037 *

Calculated Pearson’s correlation coefficient between cortisol in the blood serum and gene expression in the jejunum of chickens subjected to different challenges. * Significant correlation at *p* < 0.05. Abbreviations: *HSP70*, heat shock protein 70; *TFF2*, trefoil factor 2; *PPARα*, peroxisome proliferator-activated receptor alpha; *PPARγ*, peroxisome proliferator-activated receptor gamma; *PPARδ*, peroxisome proliferator-activated receptor delta; *P53*, tumor protein 53; *ZO-2*, zonula occludens 2; *ZO-1*, zonula occludens 1; *TLR4*, toll-like receptor 4; *OCLD*, occludin; *MUC-2*, mucin 2; *MUC-5B*, mucin 5B; *JAM-2*, junctional adhesion molecule 2; *IAP*, intestinal alkaline phosphatase; *E-cad*, e-cadherin; *CLDN-3*, claudin-3; *GLP-2*, glucagon like peptide 2; *OGG-1*, 8-oxyguanine DNA glycosylase; *GPX-1*, glutathione peroxidase 1; *CLDN-1*, claudin 1; *CD36*, CD36 molecule; *CLDN*, claudin; *CNR1*, cannabinoid receptor 1.

**Table 4:** Correlations between gene expression in the jejunum and endotoxin (LPS) concentration in the blood serum.

Gene	Endotoxin	
	*r*	*p*-Value
*HSP70*	−0.234	0.131
*TFF2*	0.045	0.769
*PPARα*	0.099	0.511
*PPARγ*	−0.154	0.308
*PPARδ*	−0.133	0.384
*P53*	0.001	1.000
*ZO-2*	−0.027	0.867
*ZO-1*	0.053	0.731
*TLR4*	0.07	0.646
*OCLN*	0.043	0.780
*MUC-2*	0.042	0.783
*MUC-5B*	0.066	0.667
*JAM-2*	−0.203	0.198
*IAP*	−0.092	0.546
*E-cad*	−0.152	0.325
*CLDN-3*	0.117	0.451
*GLP-2*	−0.255	0.091
*OGG-1*	0.21	0.177
*GPX-1*	−0.093	0.586
*CLDN-1*	−0.344	0.022 *
*CD36*	−0.325	0.028 *
*CLDN*	−0.076	0.631
*CNR1*	0.097	0.525

Calculated Pearson’s correlation coefficient between endotoxin in the blood serum and gene expression in the jejunum in chickens subjected to different challenges. * Significant correlation at *p* < 0.05. Abbreviations: *HSP70*, heat shock protein 70; *TFF2*, trefoil factor 2; *PPARα*, peroxisome proliferator-activated receptor alpha; *PPARγ*, peroxisome proliferator-activated receptor gamma; *PPARδ*, peroxisome proliferator-activated receptor delta; *P53*, tumor protein 53; *ZO-2*, zonula occludens 2; *ZO-1*, zonula occludens 1; *TLR4*, toll-like receptor 4; *OCLD*, occludin; *MUC-2*, mucin 2; *MUC-5B*, mucin 5B; *JAM-2*, junctional adhesion molecule 2; *IAP*, intestinal alkaline phosphatase; *E-cad*, e-cadherin; *CLDN-3*, claudin-3; *GLP-2*, glucagon like peptide 2; *OGG-1*, 8-oxyguanine DNA glycosylase; *GPX-1*, glutathione peroxidase 1; *CLDN-1*, claudin 1; *CD36*, CD36 molecule; *CLDN*, claudin; *CNR1*, cannabinoid receptor 1.

**Table 5:** Division into experimental groups.

Total of 204 Broiler Ross 308			
Group	Number of Birds	Additives	Challenge
CON	34	None	None
CBD	34	30 g/kg CBD	None
*C. perfringens*	34	None	*C. perfringens*
LPS	34	None	*E. coli* LPS
CBD + *C. perfringens*	34	30 g/kg CBD	*C. perfringens*
CBD + LPS	34	30 g/kg CBD	*E. coli* LPS

**Table 6:** Genes and primer sequences used in the study.

Gene	Primer	Sequence (5′-3′)	Product Size (nt)	GenBank Accession No.
*ACTB*	Forward	CGGACTGTTACCAACACCCA	115	NM_205518
	Reverse	TCCTGAGTCAAGCGCCAAAA		
*GADPH*	Forward	GCACGCCATCACTATCTT	82	NM_204305
	Reverse	GGACTCCACAACATACTCAG		
*HSP70*	Forward	GGCAATAAGCGAGCAGTG	146	NM_001006685
	Reverse	CGAGTGATGGAGGTGTAGAA		
*TFF2*	Forward	ACTACCCTACTGAGAGAACAAA	143	XM_416743
	Reverse	CTGAAGAACCTGCTCAACTG		
*PPARα*	Forward	CGGAGTACATGCTTGTGAAGG	198	XM_025150258.2
	Reverse	TCAGACCTTGGCATTCGTCC		
*PPARγ*	Forward	GACCTTAATTGTCGCATCCA	130	XM_025154399
	Reverse	TCTCCTTCTCCGCTTGTG		
*PPARδ*	Forward	TACACCGACCTTTCGCAGAG	108	NM_204728.2
	Reverse	TCCACAGACTCTGCACTCCA		
*P53*	Forward	AGGTGGGCTCTGACTGTA	98	NM_001407269.1
	Reverse	TGTAAGGATGGTGAGGATGG		
*ZO-2*	Forward	CCTCCTACCAGACCTTACC	153	NM_204918
	Reverse	CCAGCAAGCCTACAGTTC		
*ZO-1*	Forward	TCGCTGGTGGCAATGATGTT	89	XM_413773
	Reverse	TTGGTCTCCTTCCTCTAATCCTTCTT		
*TLR4*	Forward	CAAGCACCAGATAGCAACA	146	FJ915527
	Reverse	CACTACACTACTGACAGAACAC		
*OCLN*	Forward	ATCAACGACCGCCTCAAT	86	XM_046904540.1
	Reverse	TACTCCTCTGCCACATCCT		
*MUC-2*	Forward	ATCGTGAGGAATGTGAGAAGTT	140	XM_421035
	Reverse	GCAGAGGCAGAAGGAGTC		
*MUC-5B*	Forward	TGACTGTACCTGCTGCCAAG	145	XM_046919157.1
	Reverse	TGCTTCAAGGGTTTGTGGGT		
*JAM-2*	Forward	TCCTCCCACTACTCCAATATG	134	XM_026849998
	Reverse	ACTGCCTGTTCCTGTCTT		
*IAP*	Forward	CAGGAGCAGCACTATGTTG	199	XM_015291489
	Reverse	CTAGAGGAGGGCTTGGTAG		
*E-cad*	Forward	GGATGGCGTCGTCTCAACA	75	NM_001039258
	Reverse	TCCTGTGCGTAGATGGTGAAG		
*CLDN-3*	Forward	CGTCATCTTCCTGCTCTC	87	NM_204202
	Reverse	AGCGGGTTGTAGAAATCC		
*GLP-2*	Forward	TGTGTTCAGACGGTAAGG	127	NM_001163248
	Reverse	TCATCCAGTGCCATCTTC		
*OGG-1*	Forward	GAGTCTGAGTCTGGAGCA	79	XM_046926490.1
	Reverse	CTTCCTGGCTTGGCTTATC		
*GPX-1*	Forward	AGTAAAGGAAAGCCCGCACC	157	NM_001277853.3
	Reverse	GCTGTTCCCCCAACCATTTC		
*CLND-1*	Forward	GGTGAAGAAGATGCGGATG	99	NM_001013611
	Reverse	GCCACTCTGTTGCCATAC		
*CD36*	Forward	AGACCAGTAAGACCGTGAAG	134	NM_001030731
	Reverse	TAGGACTCCAGCCAGTGT		
*Tac1*	Forward	CCGATGACCTCAGCTACTGG	99	XM_004939318.3
	Reverse	GTCTCCTTGCCATCCTCTGC		
*CNR1*	Forward	GTCACCAGCGTCCTCTTG	127	NM_001038652
	Reverse	CTCCGTACTCTGAATGATTATGC		
*CNR2*	Forward	AACTGAATGAGGCTCTTCCA	194	XM_025143151
	Reverse	GCTCTTGTCACTTACTGCTG		

Abbreviations: *ACTB*, β-actin; *GADPH*, glyceraldehyde-3-phosphate dehydrogenase; *HSP70*, heat shock protein 70; *TFF2*, trefoil factor 2; *PPARα*, peroxisome proliferator-activated receptor alpha; *PPARγ*, peroxisome proliferator-activated receptor gamma; *PPARδ*, peroxisome proliferator-activated receptor delta; *P53*, tumor protein 53; *ZO-2*, zonula occludens 2; *ZO-1*, zonula occludens 1; *TLR4*, toll-like receptor 4; *OCLD*, occludin; *MUC-2*, mucin 2; *MUC-5B*, mucin 5B; *JAM-2*, junctional adhesion molecule 2; *IAP*, intestinal alkaline phosphatase; *E-cad*, e-cadherin; *CLDN-3*, claudin-3; *GLP-2*, glucagon-like peptide 2; *OGG-1*, 8-oxyguanine DNA glycosylase; *GPX-1*, glutathione peroxidase 1; *CLDN-1*, claudin 1; *CD36*, CD36 molecule; *Tac1*, tachykinin precursor 1; *CNR1*, cannabinoid receptor 1; *CNR2*, cannabinoid receptor 2.

## Data Availability

All data generated during the study are available from the corresponding author upon reasonable request.
